# Comparison of clinical indicators between perimenopausal and postmenopausal women with cardiovascular disease

**DOI:** 10.3389/fendo.2025.1681941

**Published:** 2026-01-13

**Authors:** Jing Wang, Baolin Guo, Haifeng Liu, Zhaoxia Wang, Rutao Wang, Tao Yin, Xiaoqi Liu, Xing Qin, Zhiyong Yin

**Affiliations:** 1 Department of Cardiology, Xijing Hospital, The Fourth Military Medical University, Xi’an, China; 2 The First Clinical Medical College, Shanxi Medical University, Taiyuan, Shanxi, China; 3 Department of Gynecology, First Hospital of Shanxi Medical University, Taiyuan, Shanxi, China

**Keywords:** perimenopausal, postmenopausal, cardiovascular disease, obesity, blood pressure

## Abstract

**Background:**

Cardiovascular disease (CVD) prevalence increases with age, particularly in perimenopausal and postmenopausal women due to declining estrogen levels increasing CVD risk.

**Methods and results:**

From 1248 women, 276 with CVD were screened. Clinical indicators (obesity, blood pressure, BMD, hormones) were compared between perimenopausal and postmenopausal women and across age groups (40-44, 45-49, 50-54). Postmenopausal women had significantly higher BMI, WC/HC, BFP, BFD, SBP, DBP, E2, P, PRL (*p* < 0.001), but lower FSH, LH, BMD T1/T2 (*p* < 0.001) than perimenopausal women. Significant differences in obesity indicators, BMD, and hormones existed between the 45–49 and 50–54 age groups (*p* < 0.05).

**Conclusions:**

This study elucidates how hormonal changes affect CVD development in women, providing a basis for early screening and intervention strategies.

## 1 Introduction

Cardiovascular disease (CVD) is a global chronic disease and one of the leading causes of death and disease burden worldwide (1). According to the World Health Organization (WHO), CVD cause about 17.9 million deaths each year, accounting for 32% of the total global deaths (2). The probability of CVD in the elderly is much higher than that in the young, because of the cumulative effect of increased arteriosclerosis, decreased heart function, dyslipidemia and chronic inflammation with age (3). And CVDs such as hypertension, coronary heart disease, arrhythmia and heart failure are more common in the elderly.

Women in perimenopause and postmenopause have a higher risk of CVD than men of the same age, and the reason is that the estrogen level of women in this stage decreases significantly (4). Estrogen can reduce the risk of atherosclerosis by reducing low-density lipoprotein (LDL) and increasing high-density lipoprotein (HDL) through anti-inflammatory and lipid-regulating effects (5). In addition, the reduction of estrogen increases vascular stiffness which can lead to increased blood pressure and increase the risk of hypertension and its related cardiovascular complications (6). Women in perimenopause and postmenopause are more likely to experience metabolic disorders, which can also increase the incidence of cardiovascular diseases, such as insulin resistance, abdominal obesity and elevated blood sugar (7). Especially after estrogen levels drop, women’s blood viscosity increases and coagulation factor levels rises which may cause blood clots to form in the body to increase the risk of myocardial infarction and stroke (8).

Obesity is a common risk factor for CVD, and a large amount of clinical and epidemiological evidence have shown that obesity is associated with various CVD diseases, such as coronary heart disease, heart failure, hypertension, atrial fibrillation, arrhythmia and sudden cardiac death (9–11). The maladaptation of adipose tissue expansion during the occurrence of obesity can lead to local inflammation, hypoxia, dysregulation of adipose factor secretion, impaired mitochondrial function, systemic pro-inflammatory and pro-thrombotic effects, which are all signs of CVD (12). A multivariate Cox proportional hazards regression model study on obesity and atrial fibrillation found that 13.1% of subjects developed atrial fibrillation during an average follow-up of 10.7 years, and the incidence of atrial fibrillation in obese and severely obese subjects was 13% and 32% higher than that in the normal weight group (13). There is increasing epidemiological evidence that obesity is a risk factor for deep vein thrombosis (DVT) and thrombophlebitis. Studies have found that the risk of venous thrombosis increases in a dose-dependent manner with increasing BMI and is associated with most other anthropometric indicators of overweight and obesity (14–16).

Other factors also affect the probability of CVD, such as blood pressure (17), bone density (18) and hormone levels (19). Hypertension is the main risk factor for CVD such as atherosclerosis, coronary heart disease, heart failure and stroke (20). Long-term high blood pressure can lead to vascular damage, increased heart burden, and ultimately cause serious cardiovascular events. Studies have found that the prevalence of hypertension in postmenopausal women is 2–3 times higher than that before menopause, which greatly increases the probability of CVD in postmenopausal and perimenopausal women (21). Postmenopausal and perimenopausal women experience accelerated bone density loss due to decreased estrogen levels (22). Studies have found that bone loss is fastest in the first 5–10 years after menopause, and can decrease by 1%-2% per year on average (22). In addition, the incidence of coronary heart disease and hypertension in postmenopausal women increases significantly which is positively correlated with decreased bone density. The most typical feature of postmenopausal and perimenopausal women is the significant decrease in estrogen and progesterone levels, which can cause metabolic disorders in women and increase the risk of CVD (23).

In this study, we used 176 perimenopausal women with CVD and 120 postmenopausal women with CVD as research subjects, and compared the obesity, blood pressure, bone density and hormone levels of perimenopausal and postmenopausal women with CVD at different ages to explore the differences in cardiovascular disease risk factors between perimenopausal and postmenopausal women. This study provides guidance for the clinical formulation of precise prevention and treatment measures by exploring the susceptibility factors of CVD in perimenopausal and postmenopausal women.

## 2 Methods

### 2.1 Study participants

The subjects of this study were 1248 perimenopausal and postmenopausal women aged 40 to 54 years who were registered in Xijing Hospital, Shaanxi Province, China from March 2018 to March 2024. The inclusion criteria of this study were: (1) Women aged 40 to 54 years. (2) Perimenopausal and postmenopausal women. According to WHO standards, perimenopause is defined as irregular menstrual cycles (≥7 days) or within 1–2 years before the last menstrual period (24), and postmenopause must have been natural amenorrhea for more than 12 months (25). (3) CVD patients. CVD patients must be identified through clinical symptoms, physical examination, laboratory tests, electrocardiogram (ECG) and imaging examinations, and the examination criteria must meet the examination criteria for CVD patients specified by the WHO (26). The exclusion criteria for the study subjects were: (1) Severe underlying diseases, such as advanced cancer, end-stage renal disease and active autoimmune diseases. (2) Acute cardiovascular events, such as acute myocardial infarction, acute stroke, and acute heart failure within 3 months before the start of the study. (3) Severe liver function abnormalities. (4) Habits of smoking and drinking. (5) Long-term use of hormone replacement therapy, such as estrogen drugs, anticoagulants (such as warfarin) or anti-tumor drugs.

This study was approved by the Ethics Committee of Shaanxi Xijing Hospital, and all procedures involving human subjects were in accordance with ethical standards. All participants signed written informed consent.

### 2.2 CVD detection

CVD is one of the most common metabolic diseases in postmenopausal and perimenopausal women (23). Two researchers preliminarily judged whether the subjects had CVD based on the clinical characteristics of CVD, such as chest pain or chest tightness, dyspnea, palpitations, dizziness, syncope, lower limb edema, limb numbness, and slurred speech. Patients who met the clinical characteristics of CVD needed to undergo further clinical testing according to different CVD diseases. All subjects needed to undergo blood pressure measurement and heart sound examination. Patients with systolic blood pressure (SBP) ≥140 mmHg or diastolic blood pressure (DBP) ≥90 mmHg were considered to have hypertension (27). Auscultation revealed murmurs indicating that the subjects may have valvular disease, and gallop rhythm indicating that the subjects may have heart failure. Laboratory measurements of the subjects included troponin I/T, creatine kinase isoenzyme (CK-MB), total cholesterol (TC), LDL-C, HDL-C, triglycerides (TG), and fasting blood glucose (FPG). Further testing subjects needed to undergo electrocardiograms and imaging examinations. Imaging examinations included coronary angiography, CT coronary angiography and cardiac ultrasound.

### 2.3 Body index measurement

The height of all subjects was measured using a standard stadiometer, with the subjects standing upright, barefoot, and with their heads level. The weight of the subjects was measured in the morning under a fasting state using a calibrated electronic scale, with the subjects wearing light clothing and barefoot. Body mass index (BMI) was calculated as the ratio of weight (kg) to height (m²) squared. The Chinese obesity working group (WGOC) defines people with a BMI greater than 24 as obese, and those with a BMI less than 18 as underweight (28). The waist circumference (WC) of the subjects was measured at the end of a gentle expiration with a tape measure at the midpoint between the 10th rib and the iliac crest. The hip circumference (HC) was measured with a tape measure at the widest part of the hips. The waist-to-hip ratio (WC/HC) was then calculated as the ratio of waist circumference to hip circumference. All measurements were performed by trained staff, and each parameter was measured twice to ensure accuracy.

### 2.4 Blood pressure, body fat and bone density measurement

The blood pressure of the subjects was measured using an electronic sphygmomanometer or a mercury sphygmomanometer on the right arm of the subject. Systolic blood pressure (SBP) and diastolic blood pressure (DBP) were recorded and the average value was taken for three measurements. SBP greater than 140 mmHg or DBP greater than 90 mmHg can be considered as a patient with hypertension (29). Dual-energy X-ray absorptiometry (DXA) (30) was used to measure the total body fat percentage (TBFP), total body fat distribution (TBFD), bone mineral density (BMD) and T_1_ value of the subjects’ lumbar spine L2 to L4 (L2-L4), and BMD and T_2_ value of the subjects’ femoral neck. According to the WHO standards, t-value between -1 and -2.5 is considered to be bone loss, while t-value below -2.5 is diagnosed as osteoporosis (31).

### 2.5 Blood biochemical indicators measurement

Medical staff used a syringe and needle to puncture the subject’s vein and collected 10mL of blood samples from each subject in a fasting state. After the fasting blood sample collection was completed, all subjects took 75g of glucose orally, and 10mL of blood samples were collected again by the same method 120 minutes later. The glucose oxidase method was used to measure fasting blood glucose (FPG) and blood glucose levels after oral glucose (PBG). In addition, chemical analysis was used to measure the concentrations of total cholesterol (TC), triglycerides (TG), high-density lipoprotein (HDL) and low-density lipoprotein (LDL) in the blood. Fasting blood samples were used by menopausal women to detect blood sex hormone levels, follicle-stimulating hormone (FSH), luteinizing hormone (LH), estradiol (E_2_), testosterone (T), progesterone (P) and prolactin (PRL) by enzyme-linked immunosorbent assay (ELISA) (32). Non–HDL-C was calculated as TC minus HDL. LDL-C was estimated using the Friedewald formula (33), LDL-C = TC − HDL − TG/2.2 (mmol/L). The collection of sex hormone blood samples for perimenopausal women was scheduled on the third day of the menstrual cycle, or in the case of amenorrhea for 6 months or more or more than 3 cycles.

### 2.6 Statistical analysis

Data were analyzed using SPSS 21.0 (34) (Statistical Package for Social Sciences, Inc., Chicago, IL, USA), and plotted using the *ggplot2* (35) package in R. Normally distributed data were expressed as mean ± standard deviation (SD), and skewed data were expressed as median (interquartile range). The chi-square test was used to assess the significant differences between the groups, and *p* values ​​less than 0.05 were considered significant.

## 3 Results

### 3.1 Basic information of the subjects

In this study, a total of 1248 postmenopausal and perimenopausal females were obtained from Xijing University, and 972 cases were excluded (**Supplementary Table S1**). Finally, there were 276 postmenopausal and perimenopausal female CVD patients who met the screening requirements, and the probability of CVD patients was 22.12%. This result showed that the probability of CVD in postmenopausal and perimenopausal females is much higher than that in the normal population. The number of perimenopausal women with CVD was 153, while the number of postmenopausal women with CVD was 121 (**Table 1**). The number of CVD patients in perimenopausal women aged 40-44, 45–49 and 50–54 was 37, 48 and 68, while the number of postmenopausal women was 24, 41 and 56. This result showed that the probability of CVD in perimenopausal and postmenopausal females increases with age.

**Table 1:** The number of postmenopausal and perimenopausal female with CVD at different ages.

Age	Perimenopausal women	Postmenopausal women	Total
40-44	37	24	61
45-49	48	41	89
50-54	68	56	124
Total	153	121	274

This study also compared the clinical indicators of postmenopausal and perimenopausal females with CVD to explore the differences between postmenopausal and perimenopausal females with CVD (**Table 2**). The study found that the BMI, WC/HC, TBFP and TBFD, SBP and DBP of postmenopausal females were significantly higher than those of perimenopausal females, which indicated that the probability of obesity in postmenopausal females with CVD was higher than that of perimenopausal females with CVD. The T_1_ and T_2_ values ​​of bone density in postmenopausal females with CVD were significantly lower than those in perimenopausal women with CVD, which indicated that perimenopausal women may be more susceptible to osteoporosis. There were no significant differences in blood lipid levels such as TC, TG, HDL, non-HDL-C, LDL and LDL-C between postmenopausal and perimenopausal women with cardiovascular disease, suggesting that blood lipid levels may not be the main driving factor for the differences in cardiovascular disease between the two groups. There were no significant differences in blood lipid and blood glucose indicators between postmenopausal and perimenopausal females with CVD. In terms of hormone levels, FSH and LH in perimenopausal females with CVD were significantly higher than those in postmenopausal females, while E_2_, P, and PRL were significantly lower than those in postmenopausal females. This result indicates that there are great differences in clinical indicators between postmenopausal and perimenopausal females with CVD, which may be an important influencing factor for women’s disease.

**Table 2:** Comparison of clinical parameters between postmenopausal and perimenopausal women with CVD.

Index	Perimenopausal women	Postmenopausal women	*P* value
BMI (kg/m2)	22.83 ± 3.94	24.82 ± 3.98	0.000***
WC (cm)	74.82 (65.71-83.92)	76.59 (68.19-84.98)	0.037*
HC (cm)	91.89 (85.04-98.73)	90.56 (84.79-96.33)	0.084
WC/HC	0.81 (0.76-0.87)	0.84 (0.78-0.91)	0.000***
TBFP	27.09 (21.68-32.49)	29.87 (24.68-35.07)	0.000***
TBFD	112.28 (81.54-143.02)	126.08 (99.76-152.4)	0.000***
L2-L4 (BMD)	1.06 ± 0.18	1.18 ± 0.24	0.000***
T_1_	-0.73 ± 1.29	-0.01 ± 1.42	0.000***
Neck of femur (BMD)	0.84 ± 0.15	0.93 ± 0.19	0.000***
T_2_	-0.65 ± 0.81	-0.2 ± 0.87	0.000***
TC (mmol/L)	4.99 ± 0.81	4.80 ± 0.87	0.069
TG (mmol/L)	1.31 ± 0.67	1.29 ± 0.78	0.452
HDL (mmol/L)	1.68 ± 0.41	1.63 ± 0.40	0.300
non–HDL-C (mmol/L)	3.31 ± 0.75	3.17 ± 0.82	0.153
LDL (mmol/L)	2.85 ± 0.72	2.78 ± 0.78	0.337
LDL-C (mmol/L)	2.59 ± 0.78	2.72 ± 0.73	0.168
FPG (mmol/L)	5.3 (4.49-6.11)	5.29 (4.64-5.95)	0.543
OGTT (mmol/L)	6.58 (4.63-8.53)	6.69 (5.08-8.31)	0.371
FSH (IU/L)	81.85 (49.98-113.73)	48.85 (12.64-85.07)	0.000***
LH (IU/L)	39.93 (25.56-54.3)	29.34 (6.1-52.57)	0.000***
E_2_ (pg/ml)	27.94 (-1.1-56.98)	72.09 (2.19-141.98)	0.000***
P (mmol/L)	0.38 (-0.21-0.96)	1.06 (-1.14-3.26)	0.000***
T (mmol/ml)	0.36 (-0.05-0.77)	0.4 (0.16-0.64)	0.407
PRL (ug/L)	7.97 (3.05-12.9)	10.7 (3.82-17.57)	0.000***
SBP (mmHg)	116.37 (103.72-129.02)	118.71 (106.64-130.79)	0.000***
DBP (mmHg)	76.21 (66.64-85.79)	78.19 (68.91-87.47)	0.000***

Normally distributed data were expressed as means ± SD, skewed distribution were reported as median (interquartile range), * indicated that *p* is less than 0.05, *** indicated that *p* is less than 0.001.

### 3.2 Comparison of obesity indicators in perimenopausal and postmenopausal females with CVD

**Figure 1:**
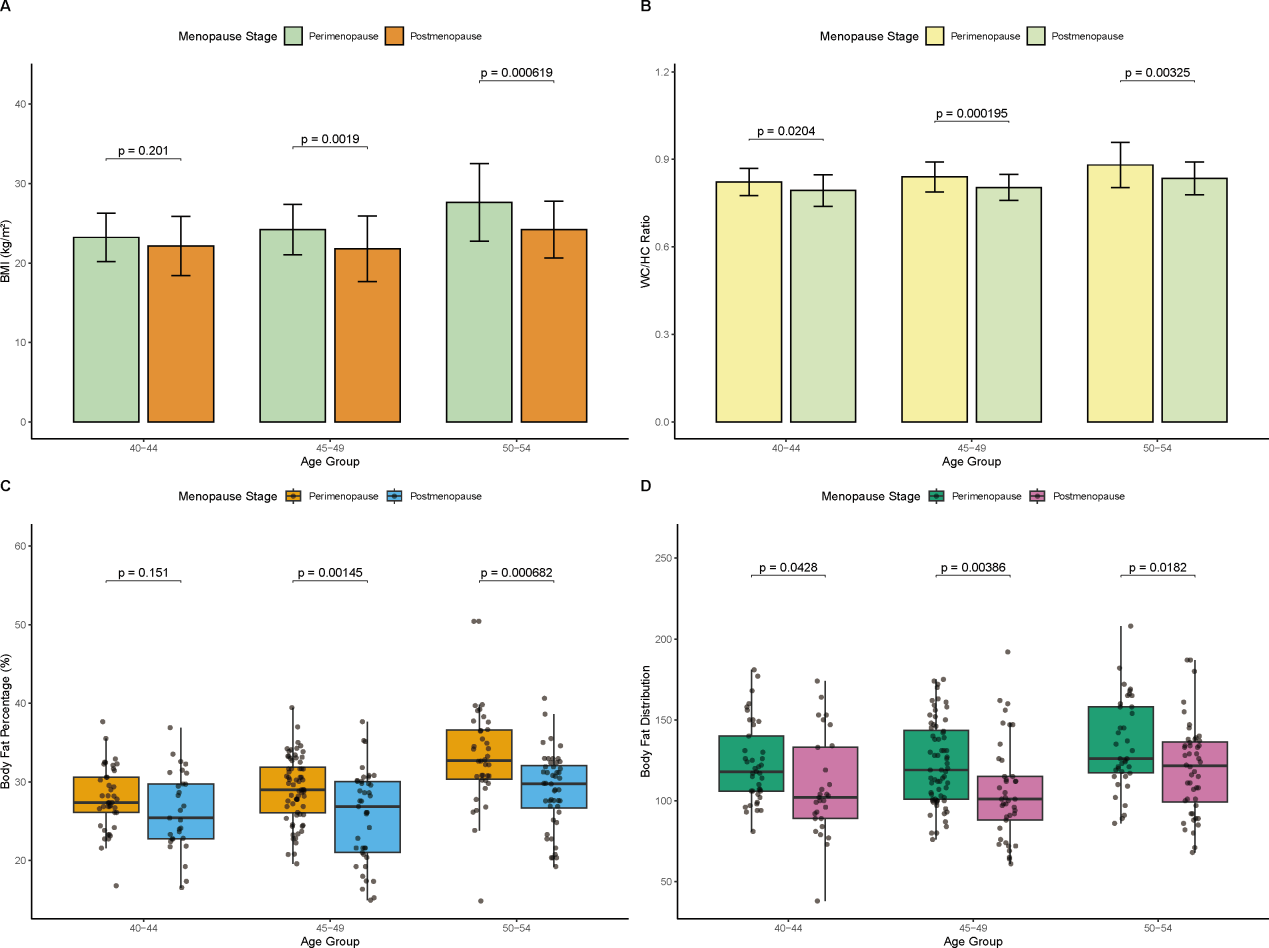
Comparison of obesity indicators in perimenopausal and postmenopausal females with CVD at different ages. **(A)** BMI. **(B)** WC/HC ratio. **(C)** Body fat percentage. **(D)** Body fat distribution.

This study compared the obesity indicators of perimenopausal and postmenopausal females with CVD at different ages to explore the impact of obesity on female CVD (**Figure 1**). The study found that the BMI value (**Figure 1A**), WC/HC ratio (**Figure 1B**), body fat percentage (**Figure 1C**) and body fat distribution (**Figure 1D**) of perimenopausal females with CVD aged 45–49 and 50–54 were significantly lower than those of postmenopausal females (*p* < 0.01). In addition, the above indicators of perimenopausal women aged 40–44 were also lower than those of the menopausal group.

In addition, this study counted the number of obese women with perimenopausal and postmenopausal CVD according to the obesity standards set by the WHO (**Supplementary Table S2**). Among perimenopausal CVD women, the number of obese people in the 40–44, 45–49, and 50–54 age groups were 16, 23 and 49, while among postmenopausal CVD women, the number of obese people in different age groups was 9, 13 and 30, respectively. Overall, the obesity rate in perimenopausal women was 57.52%, and that in postmenopausal women was 47.89%. This result indicates that obesity is more common in perimenopausal women with CVD, and the hormone level fluctuations and metabolic disorders of women at this stage aggravate fat accumulation at this stage, thereby increasing the risk of cardiovascular disease.

### 3.3 Comparison of blood pressure in perimenopausal and postmenopausal females with CVD

**Figure 2:**
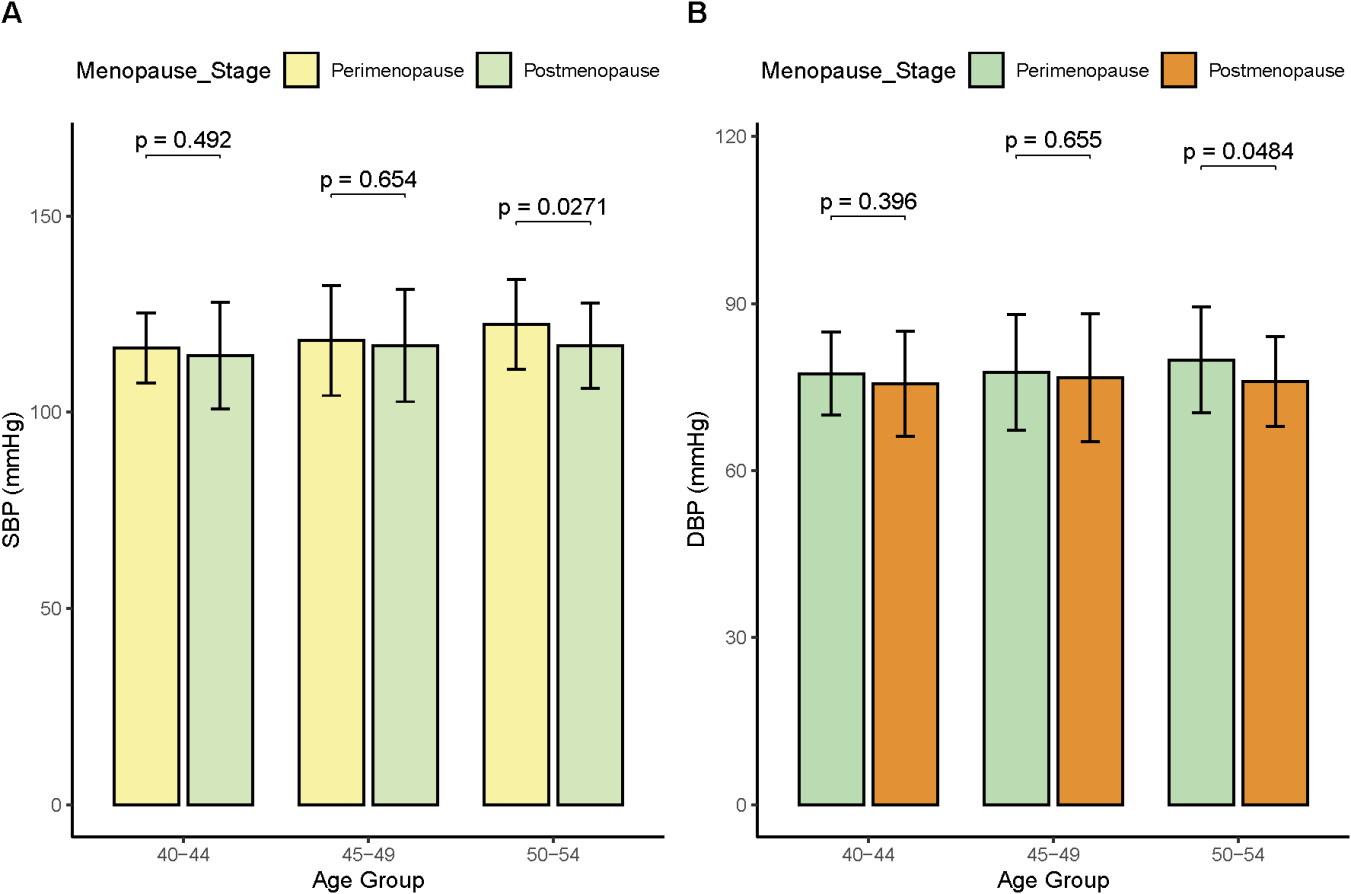
Comparison of blood pressure in perimenopausal and postmenopausal females with CVD at different ages. **(A)** SBP. **(B)** DBP.

This study also compared the blood pressure of perimenopausal and postmenopausal females with CVD at different ages (**Figure 2**). The SBP (**Figure 2A**) and DBP (**Figure 2B**) of perimenopausal females aged 40–44 and 45–49 were slightly higher than those of postmenopausal females, but there was no significant difference (*p* > 0.05). However, the SBP and DBP of perimenopausal females aged 50–54 were significantly higher than those of postmenopausal females (*p* < 0.05). We also conducted statistics on the hypertensive population of perimenopausal and postmenopausal females with CVD (**Supplementary Table S3**). The number of perimenopausal females with hypertension at 40-44, 45–49 and 50–54 was 11, 24 and 32, while the number of postmenopausal women at different age stages was 7, 11 and 24. The proportion of hypertension in perimenopausal females with CVD was 41.83%, while the proportion of hypertension in postmenopausal women with CVD was 34.71%. This result shows that the probability of perimenopausal females suffering from hypertension is significantly higher than that of postmenopausal women.

### 3.4 Comparison of hormone level in perimenopausal and postmenopausal females with CVD

**Figure 3:**
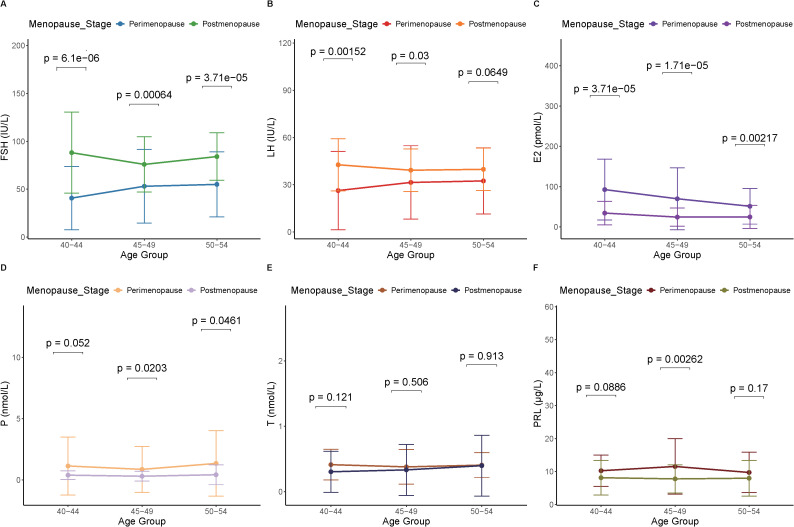
Comparison of hormone level in perimenopausal and postmenopausal females with CVD at different ages. **(A)** Follicle-stimulating hormone (FSH). **(B)** Luteinizing hormone (LH). **(C)** Estradiol (E_2_). **(D)** Progesterone (P). **(E)** Testosterone (T). **(F)** Prolactin (PRL).

This study also compared the levels of six hormones in perimenopausal and postmenopausal females with CVD, FSH, LH, E2, P, T and PRL (**Figure 3**). The study found that the hormone levels of FSH and E2 in postmenopausal females with CVD at 40-44, 45–49 and 50–54 were significantly higher than those in perimenopausal females with CVD (*p* < 0.05). The hormone level of LH in postmenopausal females with CVD at 40–44 and 50–54 was significantly higher than that in perimenopausal females with CVD (*p* < 0.05). The hormone levels of P in postmenopausal females with CVD at 45–49 and 50–54 were significantly lower than those in perimenopausal females with CVD (*p* < 0.05), while the hormone levels of T and PRL in postmenopausal females with CVD at 40–44 and 50–54 were significantly lower than those in perimenopausal females with CVD, but there was no significant difference (*p* > 0.05). This result indicates that there are differences in hormone levels between perimenopausal and postmenopausal females with CVD, which may be the main factor in the difference in CVD between the two female groups.

### 3.5 Multiple regression analysis of E_2_ and other clinical indicators

**Figure 4:**
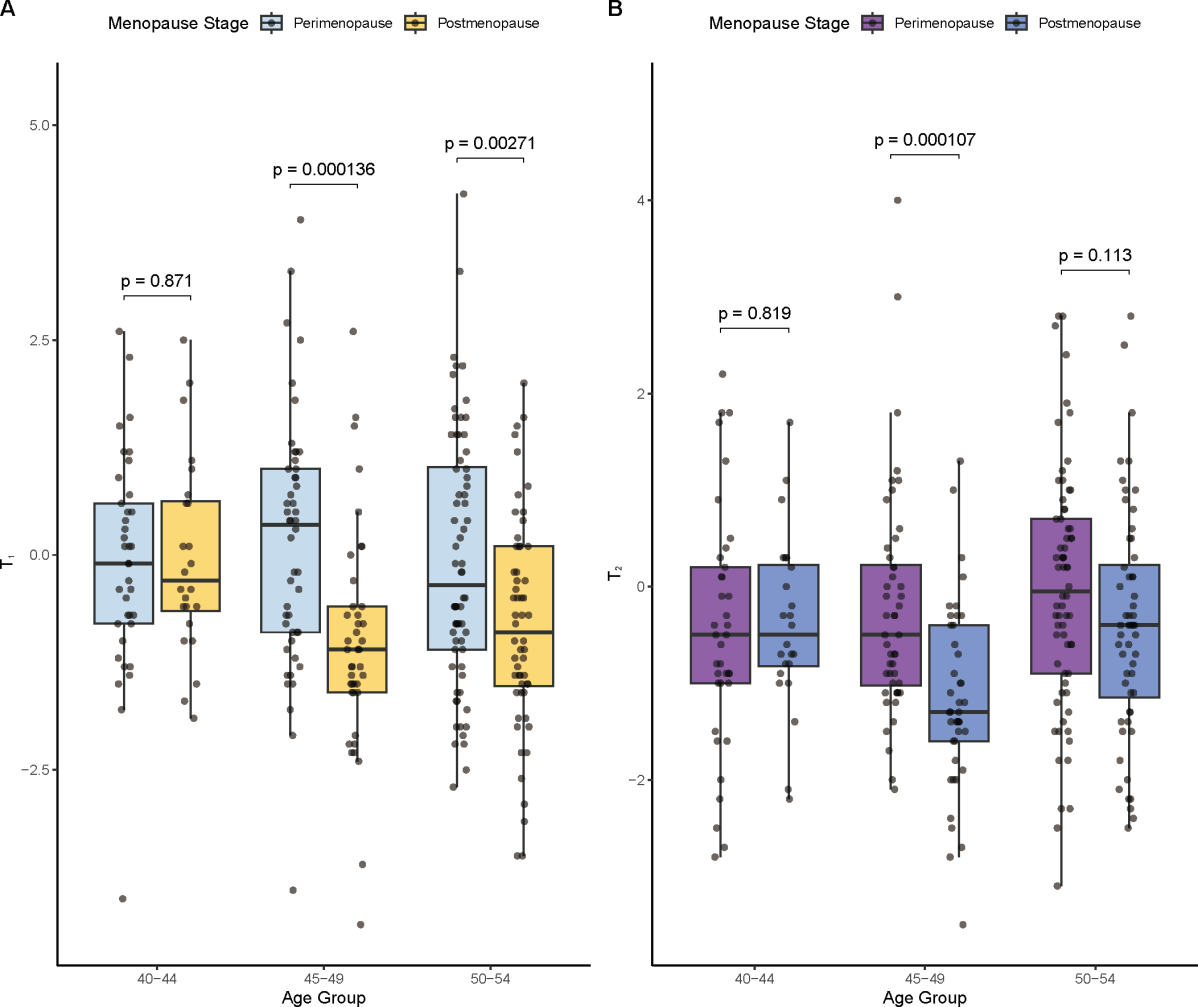
Comparison of bone mineral density in perimenopausal and postmenopausal females with CVD at different ages. **(A)** T_1_ value of L2-L4 bone mineral density. **(B)** T_2_ value of neck of femur bone mineral density.

In perimenopausal and postmenopausal women, changes in E_2_ levels are key factors affecting their health status, such as CVD, bone density and fat metabolism (**Figure 4**). Therefore, this study analyzed the correlation between other clinical indicators and E_2_ in perimenopausal and postmenopausal women (**Table 3**). The results of multiple regression analysis found that FSH was significantly negatively correlated with E_2_ (*p* < 0.001), LH and PRL were significantly positively correlated with E_2_ (*p* < 0.01). However, the regression coefficients of BMI, T_1_, T_2_ and P did not reach the significant level (*p* > 0.05), indicating that the direct association between these factors and E_2_ was weak. This result suggests that the reduction of E_2_ levels may indirectly promote the occurrence and development of CVD in menopausal women by affecting the regulatory mechanism of FSH, LH, and PRL. In addition, BMI, T_1_, T_2_, and P did not show significant correlation, indicating that these indicators may be more affected by other metabolic or environmental factors rather than directly determined by E_2_ levels.

**Table 3:** Multivariate regression analysis of E_2_ and other clinical parameters in perimenopausal and postmenopausal females with CVD.

	Estimate	Std. error	t value	Pr (>|t|)
(Intercept)	67.70622	16.10475	4.204	0.000***
BMI	-0.34121	0.53793	-0.634	0.527
T_1_	-0.3494	1.6684	-0.209	0.834
T_2_	-1.13318	2.16169	-0.524	0.601
FSH	-0.79918	0.09733	-8.211	0.000***
LH	0.55639	0.1831	3.039	0.003**
P	-2.62566	9.20272	-0.285	0.776
PRL	1.83224	0.66534	2.754	0.006 **

## 4 Discussion

This study compared cardiovascular disease-related clinical indicators in 153 perimenopausal women and 121 postmenopausal women. Results showed that postmenopausal women had significantly higher levels of obesity-related indicators (BMI, WC/HC, TBFP and TBFD), systolic and diastolic blood pressure than perimenopausal women, while their bone mineral density indicators (T_1_ and T_2_) were significantly lower. Regarding hormones, postmenopausal women had significantly lower FSH and LH levels than perimenopausal women, while their E_2_, P, and PRL levels were significantly higher. Moreover, this study also compared the differences in obesity indicators, BMD, blood pressure and hormone levels between perimenopausal and postmenopausal females with CVD at different ages, further indicating the differences in clinical indicators between perimenopausal and postmenopausal females with CVD.

### 4.1 Obesity in perimenopausal and postmenopausal females with CVD

Obesity is an important risk factor for cardiovascular health in perimenopausal and postmenopausal women (36). During the menopausal transition period and after menopause, as the estrogen level decreases, the body’s fat cell differentiation is abnormal and it is very easy to cause abdominal fat accumulation (37). With the changes in the pituitary-hypothalamus-ovarian reproductive axis (HPO axis), the ovaries age and degenerate, and the balance between sex hormones in the body has changed greatly. The production of estrogen in the body is transferred to fat cells, and a series of abnormalities in sugar and fat metabolism occur at the same time, resulting in abnormal body fat distribution, especially abdominal obesity with accumulation of intra-abdominal fat (38). In this study, BMI, WC/HC, BFP and BFD of postmenopausal women were significantly lower than those of perimenopausal women, and the probability of obesity in perimenopausal women in this study was also higher than that of postmenopausal women. This may be related to the drastic fluctuations in hormone levels and metabolic regulation disorders during this stage.

Estrogen levels decrease during perimenopausal and postmenopausal periods, which can lead to redistribution of body fat in women and greatly increase the probability of obesity (39). The main manifestation is that subcutaneous fat decreases and visceral fat increases, which can lead to abdominal obesity in perimenopausal and postmenopausal females, which greatly increases the risk of CVD. Obesity affects the structure of the heart, mainly manifested as an increase in the diameter of the left atrium and hypertrophy of the left ventricle (40). A study found through the Framingham cohort study that the left atrium of obese patients can be enlarged and the incidence of atrial fibrillation can increase (41).

### 4.2 Bone loss in perimenopausal and postmenopausal females with CVD

CVD and osteoporosis are common diseases in elderly women (42). After total oophorectomy in premenopausal women, E_2_ levels in blood and tissues decrease significantly which can cause osteoporosis. This suggests that E_2_ levels may be closely related to the occurrence and severity of osteoporosis (43). E_2_ indirectly regulates the secretion and function of corticosteroid hormones by binding to estrogen receptors in target cells and regulating the synthesis and metabolism of intracellular deoxyribonucleic acid (DNA), ribonucleic acid (RNA), and proteins. In postmenopausal women, E_2_ levels in the body decrease significantly, and the expression level of E_2_ receptors in bone tissue also decreases (44). The combined effect of the two causes bone resorption to exceed bone formation that can result in bone loss and decreased bone density to induce postmenopausal osteoporosis.

Recent studies have found that the incidence of both diseases increases significantly with age, and the degree of vascular calcification is positively correlated with the severity of osteoporosis (45, 46). Studies have found that osteoporosis patients are often accompanied by hypercholesterolemia and vascular calcification, and further studies have found that osteoporosis and hyperlipidemia are positively correlated (47). Lipid peroxidation not only exists in the vascular walls of atherosclerosis, but similar lesions can also be found in the bone tissue or blood vessels around the bone tissue of osteoporosis patients. Studies have found that lipid peroxidation can induce osteoblast differentiation to promote vascular calcification (48). Lipid peroxidation can inhibit the differentiation of osteoblasts in bones, which can lead to the simultaneous occurrence of atherosclerosis and osteoporosis.

### 4.3 Estrogen in perimenopausal and postmenopausal females with CVD

There is a close relationship between estrogen and cardiovascular health (49). Estrogen can improve endothelial function, regulate blood lipid metabolism, inhibit the activation of the coagulation system and reduce the occurrence of arteriosclerosis (50). Endothelial cells are a layer of cells on the inner wall of blood vessels, which play an important role in regulating vascular tension, maintaining vascular stability and preventing thrombosis. Estrogen can promote the synthesis and release of nitric oxide, which is an important vasodilator factor that can relax vascular smooth muscle, increase the inner diameter of blood vessels, lower blood pressure and improve cardiovascular function (51). Moreover, estrogen can also inhibit the inflammatory response and oxidative stress of endothelial cells and protect endothelial function (52).

Abnormal blood lipid metabolism is one of the important risk factors for cardiovascular disease. Estrogen can affect the synthesis and metabolism of cholesterol, reduce LDL-C levels and increase HDL-C levels (53). LDL-C is considered to be the main pathogenic factor of atherosclerosis, while HDL-C has an anti-atherosclerotic effect. In this study, we found no significant difference in blood lipid levels between perimenopausal and postmenopausal women with CVD. However, a recent study on familial hypercholesterolemia (FH) patients showed that female FH patients had a significantly lower LDL-C target achievement rate after lipid-lowering therapy compared to male patients, especially those carrying the SLCO1B1 rs4149056 gene variant (54). This suggests that menopausal status may not be a major risk factor for CVD, while genetic background and pharmacological interventions may play a crucial role in lipid regulation and cardiovascular risk management. Therefore, estrogen helps reduce the risk of cardiovascular disease by regulating blood lipid metabolism. Moreover, clinical studies have found that estrogen supplementation after menopause can effectively reduce the occurrence of cardiovascular and cerebrovascular diseases (55, 56).

In this study, we found significant differences in PRL and E_2_ levels between perimenopausal and postmenopausal women, as well as between age groups 40–44 and 45–49 years. These results suggest that hormonal changes from perimenopause to menopause exhibit distinct phased and individualized characteristics, rather than a simple linear decline. Declining E_2_ levels primarily reflect the gradual decline in ovarian function with age and menopause (57), a core hallmark in endocrinology. Declining estrogen levels alter fat distribution and increase visceral fat mass, which can increase CVD risk. Differences in PRL levels may be closely related to the remodeling of hypothalamic-pituitary-ovarian axis function (58). During perimenopause, pituitary hormone regulation and feedback mechanisms change and altered metabolic status (such as increased body fat, insulin resistance and low-grade chronic inflammation) may further influence PRL secretion (59). Furthermore, psychosocial stress and lifestyle factors indirectly regulate PRL and sex hormone levels by activating the hypothalamic-pituitary-adrenal (HPA) axis and sympathetic nervous system.

Women in perimenopause gradually experience various adverse changes in their body composition, such as elevated blood pressure, disrupted glucose metabolism, decreased hormone levels and abnormal lipid profiles (4). These changes significantly increase the risk of cardiovascular disease in middle-aged women. Many studies have proposed the timing hypothesis (60, 61) which suggests that initiating hormone therapy during perimenopause or early postmenopause may result in more favorable cardiovascular outcomes than initiating intervention after atherosclerotic lesions have matured or even progressed. Therefore, integrated management strategies should be implemented early in the aging process to effectively reduce the long-term burden of cardiovascular disease, including hormone regulation, metabolic improvement and lifestyle interventions.

Multiple studies have demonstrated that hormone replacement therapy (HRT) can significantly protect against cardiovascular disease when initiated during perimenopause or early menopause (55, 62). However, HRT often fails to reverse established atherosclerotic lesions for those initiated late (many years after menopause) (63). This late initiation is primarily due to the presence of fibrosis, lipid deposition and chronic inflammation in the vascular wall, which limits the effects of estrogen on improving vascular elasticity and hemodynamics, weakening its potential cardiovascular protective effects (64). Strategies for healthy aging in perimenopausal and postmenopausal women include regular monitoring of hormone levels and cardiovascular risk markers (such as blood pressure, blood lipids, blood sugar and BMI). Exercise, diet, weight management, and mental health interventions should be used to reduce metabolic abnormalities and cardiovascular burden. After rigorous assessment of cardiovascular and breast risk, personalized HRT can be used as an adjunct, dynamically adjusted in conjunction with lifestyle interventions to optimize long-term cardiovascular health outcomes.

This study still has some limitations. The aim of this study was to compare the clinical characteristics of perimenopausal and postmenopausal women diagnosed with CVD. Therefore, the initial analysis only included samples from confirmed CVD patients to explore the impact of menopause in the CVD population. Because samples from non-CVD women were not included in the data collection process, we were unable to conduct any supplementary analyses on healthy women. Future research should include a control group of non-CVD women to improve the gender universality and reliability of the results. And it did not collect information on lipid-lowering treatments used by participants, thus preventing the assessment of the potential impact of pharmacological interventions on lipid levels and cardiovascular risk, such as the use of statins, fibrates or other lipid-lowering drugs. Although this study included several cardiovascular disease-related clinical indicators, it failed to obtain vascular assessment data directly related to atherosclerosis, such as carotid intima-media thickness (CIMT), atherosclerotic plaque burden, ankle-brachial index (ABI) or coronary artery calcium score (CAC). Future research should incorporate more comprehensive lipid management information and vascular imaging indicators to more accurately assess the mechanisms underlying changes in cardiovascular risk in perimenopausal and postmenopausal women.

## 5 Conclusion

This study compared the clinical data of perimenopausal and postmenopausal women with CVD to find differences in their clinical indicators. Postmenopausal women had significantly higher levels of obesity-related indicators (BMI, WC/HC, TBFP and TBFD), systolic and diastolic blood pressure than perimenopausal women, while their bone mineral density indicators (T_1_ and T_2_) were significantly lower. In terms of hormones, postmenopausal women had significantly lower levels of FSH and LH than perimenopausal women, while their levels of E_2_, P and PRL were significantly higher. Moreover, regression analysis of E_2_ and BMI, BMD and other hormone levels found that E_2_ and other hormone levels were significantly correlated, which showed that estrogen levels play a key role in the health of perimenopausal and postmenopausal women. This study enhances our understanding of the relationship between hormone fluctuations and cardiovascular risk in women during the menopausal transition period, and provides a theoretical basis for the development of early screening, prevention, and intervention strategies for cardiovascular disease in perimenopausal and postmenopausal women.

## Data Availability

The raw data supporting the conclusions of this article will be made available by the authors, without undue reservation.

## Supplementary material

Supplementary Table 1 Original data of 296 perimenopausal and postmenopausal women with CVD.

Supplementary Table 2 The number of obese women with cardiovascular disease at different ages during perimenopause and postmenopause.

Supplementary Table 3 The number of hypertension women with cardiovascular disease at different ages during perimenopause and postmenopause.
